# Silencing heme oxygenase-1 increases the sensitivity of ABC-DLBCL cells to histone deacetylase inhibitor *in vitro* and *in vivo*

**DOI:** 10.18632/oncotarget.19652

**Published:** 2017-07-28

**Authors:** Zhen Zhou, Qin Fang, Dan Ma, Nana Zhe, Mei Ren, Bingqing Cheng, Peifan Li, Ping Liu, Xiaojing Lin, Sishi Tang, Xiuying Hu, Yudan Liao, Yaming Zhang, Tingting Lu, Jishi Wang

**Affiliations:** ^1^ Department of Hematology, Affiliated Hospital of Guizhou Medical University, Guiyang 550004, China; ^2^ Key Laboratory of Hematological Disease Diagnostic and Treatment Centre of Guizhou Province, Guiyang 550004, China; ^3^ Department of Hematology, Guizhou Provincial Laboratory of Hematopoietic Stem Cell Transplantation Center, Guiyang 550004, China; ^4^ Department of Pharmacy, Affiliated Hospital of Guizhou Medical University, Guiyang 550004, China; ^5^ Department of Pharmacy, Affiliated Baiyun Hospital of Guizhou Medical University, Guiyang 550004, China

**Keywords:** heme oxygenase-1, histone deacetylase 3, vorinostat, P27, diffuse large B-cell lymphoma

## Abstract

Heme oxygenase-1 (HO-1) can promote tumor growth and reinforce the resistance of diffuse large B-cell lymphoma (DLBCL) cells to chemotherapeutic drug vincristine. We herein found that HO-1 protein expression was higher in high-risk DLBCL patients than in low-risk ones. Silencing HO-1 gene expression resisted vorinostat-induced apoptosis and arrested cell cycle in the G0/G1 phase of LY-10 cells. Western blot, co-immunoprecipitation and chromatin immunoprecipitation assays confirmed that the possible mechanisms may be increased cleaved caspase-3 protein expression, decreased phospho-histone deacetylase 3 protein expression, and activated histone acetylation of P27^Kip1^ promoter. Moreover, silencing HO-1 gene expression enhanced vorinostat-induced tumor cell apoptosis, prolonged survival time and promoted P27^Kip1^ protein expression in a xenograft mouse model.

In conclusion, HO-1 is a potential therapeutic target of DLBCL. The findings provide a valuable preclinical evidence for sensitizing DLBCL patients with poor prognosis to histone deacetylase inhibitors.

## INTRODUCTION

Diffuse large B-cell lymphoma (DLBCL) is an aggressive B-cell non-Hodgkin lymphoma (NHL) that has a wide range of clinical presentations [1]. According to gene expression profiling, DLBCL can be classified into germinal-center B-cell–like DLBCL (GCB-DLBCL) and activated B-cell–like DLBCL (ABC-DLBCL) subtypes [2].

In the post-rituximab era, the first-line therapy for DLBCL is a combination of rituximab, cyclophosphamide, doxorubicin, vincristine, and prednisone (R-CHOP) [3], and the outcomes of patients with DLBCL have been substantially improved (3-year estimate of event-free survival was 67% in the R-CHOP group) [4]. However, approximately 40% of patients are refractory to treatment or relapse after receiving current standard immunochemotherapy R-CHOP [5]. These refractory or relapsed DLBCL patients survive fewer than 5 years, and they also have lower survival rates [6, 7]. Moreover, patients with ABC-DLBCL have poor outcomes compared with GCB-DLBCL patients (5-year overall survival, 35% vs. 60%; P<0.001) [8]. Therefore, it is necessary to determine a novel therapeutic approach for treating DLBCL patients with poor prognosis.

HDACis (histone deacetylase inhibitors) have well-characterized antitumor activities, also being well tolerated as a specific strategy [9]. Vorinostat (Suberoylanilide hydroxamic acid, SAHA) is a representative HDACi and has been approved to treat relapsed/refractory cutaneous T-cell NHL [10]. SAHA is effective for both T-cell and B-cell NHL cell lines [11]. The mechanisms for SAHA treatment of B-cell NHL have been explored. P27^Kip1^ protein may play an important role in HDACi-mediated tumor cell death and cell cycle arrest in the G0/G1 phase of an Emu-myc B-cell lymphoma model [11]. P27^Kip1^ is often referred to as a cell cycle inhibitor protein because it can stop or slow down the cell division cycle, and bind other cyclin-dependent kinase (CDK) proteins such as cyclin E and CDK2 [12]. Regardless of many advantages in B-cell NHL treatment, SAHA has not been approved for treating DLBCL hitherto.

Heme oxygenase (HO-1) is a well-known rate-limiting enzyme in the catabolism of heme [13], and functions as a pro-oxidant, immune regulator, and cytoprotector against inflammatory diseases [14–17]. HO-1 can also up-regulate nuclear factor-κB (NF-κB) and Nrf2 which target the upstream promoter region of HO-1 [18, 19]. HO-1 expression also increases protein phosphorylation. For example, HO-1 knockout decreases Akt phosphorylation in mice [20]. Our group has reported that HO-1 was overexpressed in ABC-DLBCL, multiple myeloma (MM), chronic myelogenous leukemia (CML), and acute myelogenous leukemia (AML). Besides, HO-1 overexpression has been related to increased tumor proliferation and resistance to chemotherapeutic drugs, probably through a negative feedback loop [18, 21–26]. Therefore, we hypothesized that HO-1 may be involved in the resistance of DLBCL to SAHA.

In this study, we demonstrated that HO-1 protein expression was positively correlated with International Prognostic Index (IPI) classification of ABC-DLBCL patients, and silencing HO-1 gene expression induced apoptosis and arrested cell cycle in the G0/G1 phase in ABC-DLBCL cell line LY-10 after SAHA treatment.

## RESULTS

### Expressions of HO-1, HDAC1, HDAC2, HDAC3 and P27^Kip1^ in DLBCL

**Figure 1:**
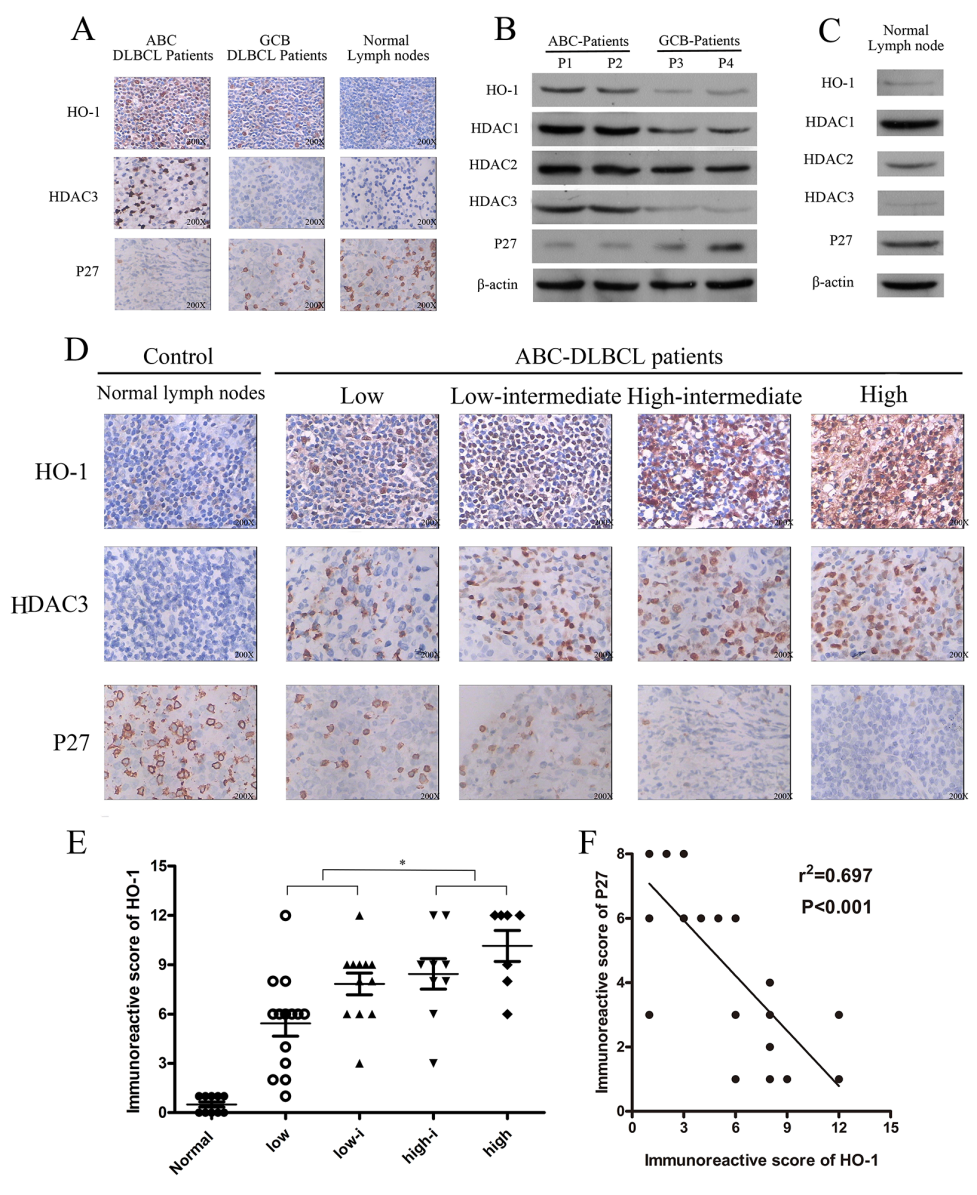
Expressions of HO-1, HDAC3 and P27 in diffuse large B-cell lymphoma (DLBCL) **(A, D)** Expressions of HO-1, HDAC3 and P27 in DLBCL tissue samples were assessed at the protein level by immunohistochemistry. A representative example (ABC-DLBCL: 42; GCB-DLBCL: 8; NLD: 5) is shown (400 ×). **(B, C)** Expressions of HO-1, HDAC1, HDAC2, HDAC3 and P27 in different DLBCL samples and NLDs were assessed at the protein relative level by Western blot. **(E)** Scatter diagrams of HO-1 protein expressions in different risk groups of ABC-DLBCL patient samples by immunoreactive scores. **(F)** Correlation between HO-1 and P27 immunoreactive scores in ABC-DLBCL patient samples (r^2^=0.697; P<0.01). All experiments were repeated three times. *P<0.05, **P<0.01. Activated B-cell-like DLBCL (ABC-DLBCL), germinal-center B-cell-like DLBCL (GCB-DLBCL), normal lymph nodes (NLD).

HO-1 and HDAC3 protein expressions were higher in ABC-DLBCL than those in GCB-DLBCL. P27^Kip1^ protein was lowly expressed in ABC-DLBCL and GCB-DLBCL samples, but positively expressed in normal lymph nodes (Table 1 and Figure 1A). HDAC3 is a target of DLBCL, and low expression of P27^Kip1^ has been correlated with poor survival (P<0.001) of DLBCL patients [27, 28]. Also, HDAC3 protein had strongly positive expression in ABC-DLBCL. However, it had negative or weakly positive expression in GCB-DLBCL and normal lymph nodes (Figure 1B-1D). Meanwhile, normal lymph nodes expressed HDAC1, HDAC2, and P27^Kip1^ proteins but not HO-1 or HDAC3 protein (Figure 1C). Therefore, HO-1 and HDAC3 were aberrantly co-expressed with P27^Kip1^ in ABC-DLBCL cells.

**Table 1:** Clinical characteristics of DLBCL patients

Parameters	No. of patients (%)
Age, median, years	56 (range, 24–79)
Age > 60	22 (44.0)
Female/male	28/22
Histopathological subtypes by GEP	
GCB subtype	8 (16.0)
ABC subtype	42 (84.0)
ECOG Performance status	
0–1	23 (46.0)
≥2	27 (54.0)
Stage	
I/II	24 (48.0)
III/IV	26 (52.0)
Extranodal involvement	
0–1	21 (42.0)
≥2	29 (58.0)
LDH	
Normal	22 (44.0)
>Normal	28 (56.0)

ABC=activated B-cell-like, ECOG=Eastern Cooperative Oncology Group, GCB=germinal center B-cell-like, GEP=gene-expression profiling, LDH= lactate dehydrogenase.

HO-1 protein expression was higher in high-risk ABC-DLBCL patients than in low-risk ones (P<0.05) (Figure 1D). In addition, HO-1 protein expression was positively correlated with IPI classification (Table 2 and Figure 1E). Moreover, HO-1 immunoreactive scores were negatively correlated with P27^Kip1^ protein expression in ABC-DLBCL patients (R^2^=0.697; P<0.01; Figure 1F).

**Table 2:** Correlation between HO-1 expression and clinic pathological features of ABC-DLBCL patients

Characteristics	No. n=42	HO-1 expression		P-value
		Immunoreactive (1-6 scores)	Immunoreactive (7-12 scores)	
Age, years; n (%)				0.358
≤60	24	12	12	
>60	18	6	12	
Gender				0.621
Female	21	8	13	
Male	21	10	11	
ECOG Performance status				0.024
<2	18	11	7	
≥2	24	7	17	
Stage				0.087
I or II	20	11	9	
III or IV	22	7	15	
Extranodal involvement				0.049
1 site	18	10	8	
>1 site	24	8	16	
LDH level				0.295
Normal	17	7	10	
High	25	11	14	
IPI				<0.01
Low to low- intermediate	26	15	11	
High-interm-ediate to high	16	3	13	

### SAHA treatment increased HO-1 gene expression in ABC-DLBCL cell line LY-10 via the NF-κB pathway

**Figure 2:**
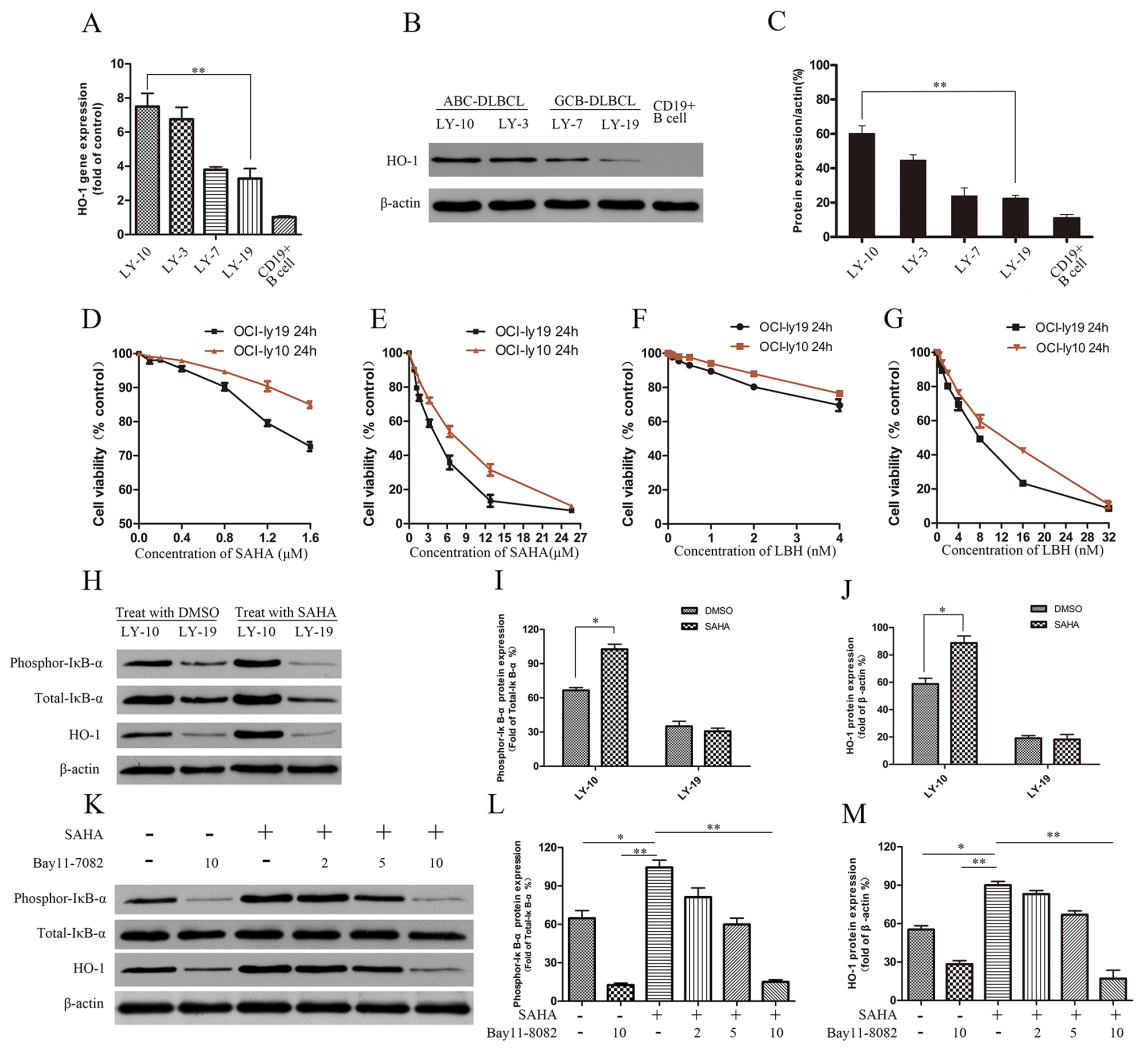
Effects of HDACis (SAHA and LBH) on DLBCL cell lines LY-10 and LY-19 **(A)** Relative HO-1 gene expressions in different DLBCL cell lines by real-time PCR. **(B, C)** HO-1 protein expressions in different DLBCL cell lines and CD19^+^ B cells were detected by Western blot. **(D-G)** LY-10 and LY-19 cells were treated with vorinostat (SAHA) (0.1–25.6 μM) and panobinostat (LBH) (0.1–32 nM). Cell proliferation inhibitions were detected with the cell-counting kit. **(H-J)** LY-10 and LY-19 cells were treated with SAHA (8 μM) or DMSO (1%). Protein expressions of HO-1, phospho-IκB-α^S32/S36^ and total-IκB-α were detected by Western blot. **(K-M)** LY-10 cells were pretreated with NF-κB inhibitor Bay11-7082 (2, 5, and 10 μM) for 1 h and then treated with or without SAHA (8 μM). Protein expressions of HO-1, phospho-IκB-α^S32/S36^ and total-IκB-α were detected by Western blot. Western blot bands were quantified with Quantity One software. Each sample was normalized by related β-actin expression or total-IκB-α expression. All experiments were repeated three times. *P<0.05, **P<0.01.

In DLBCL cell lines (ABC-DLBCL cell lines LY-3 and LY-10, GCB-DLBCL cell lines LY7 and LY-19), HO-1 expression was detected by real-time PCR and Western blot. HO-1 gene and protein expressions were higher in LY-10 cells than in LY-19 cells (Figure 2A-2C). The effects of SAHA on the proliferation of LY-10 and LY-19 cells were evaluated by counting kit-8 (CCK-8) assay. Treating LY-10 and LY-19 cells with low-concentration SAHA (0.5 μM) for 24 h did not significantly inhibit cell proliferation (Figure 2D-2G). However, silencing of HO-1 in combination with SAHA (0.5 μM) facilitated the apoptosis of LY-10 cells in the siHO-1 group compared with that of the control group (P<0.05) (Supplementary Figure 4). Similarly, Dasmahapatra et al. reported that SAHA did not induce LY-10 cell apoptosis at low concentration (0.5 μM) [29]. As shown in Figure 2H and Supplementary Figure 1, treatment of LY-10 cells with SAHA can up-regulate HO-1 and phospho-IκB-α^S32/S36^ protein expressions. Likewise, SAHA can cause NF-κB activation [29–31]. To verify whether HO-1 expression resulted from activation of NF-κB, we treated LY-10 cells with NF-κB inhibitor (Bay11-7082). Bay11-7082 inhibited HO-1 expression in LY-10 cells induced by SAHA (Figure 2K and Supplementary Figure 2). Thus, HO-1 was at the downstream of NF-κB.

### Regulation of HO-1 gene expression mediated by lentivirus in LY-10 cells

**Figure 3:**
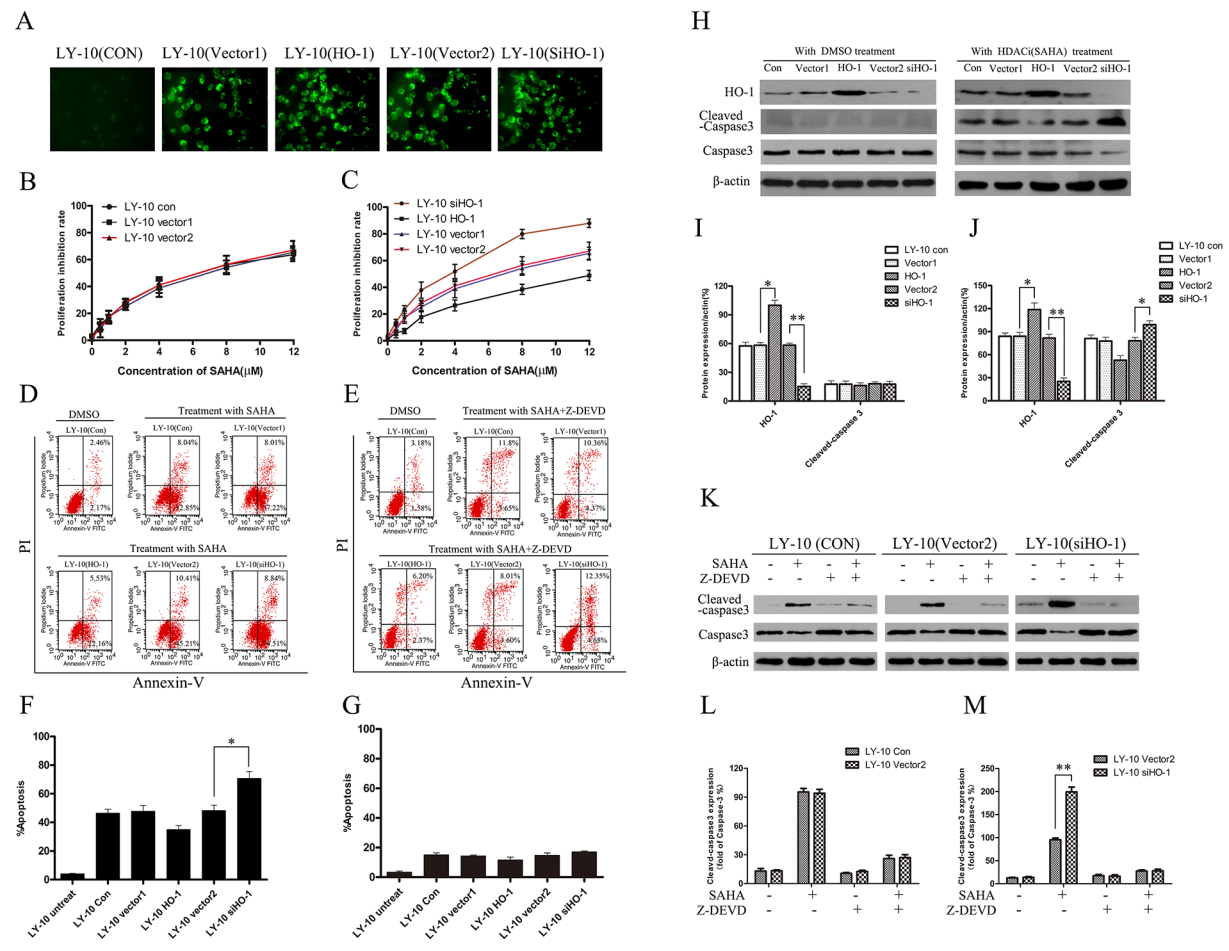
Silencing HO-1 gene expression sensitized LY-10 cells to apoptosis induced by vorinostat (SAHA) **(A)** LY-10 cells were grouped into LY-10 (control), LY-10 (vector 1), LY-10 (HO-1), LY-10 (vector 2), and LY-10 (siHO-1). The positivity of lentivirus-mediated HO-1 and siHO-1 transduction (>95%) was observed by fluorescence microscopy. **(B, C)** Different groups of LY-10 cells were treated with vorinostat (SAHA) (0.1–12 μM) for 24 h. Cell proliferation inhibitions were detected with a cell-counting kit. **(D, F)** Different groups of LY-10 cells were treated with SAHA (8 μM) or DMSO (0.1%) for 24 h. Apoptosis rate was detected by flow cytometry. **(E, G)** Different groups of LY-10 cells were treated with SAHA (8 μM) combined with caspase-3 inhibitor (Z-DEVD) (50 μM) or DMSO (0.1%) for 24 h. Apoptosis rate was detected by flow cytometry. **(H-J)** Protein expressions of HO-1, caspase-3, and cleaved caspase-3 were detected by Western blot. **(K-M)** LY-10 (control), LY-10 (vector 2), and LY-10 (siHO-1) were treated with caspase-3 inhibitor (Z-DEVD) (50 μM) with or without SAHA (8 μM). Protein expressions of caspase-3 and cleaved caspase-3 were detected by Western blot. Western blot bands were quantified with Quantity One software. Data were analyzed with Prism V5.0 (GraphPad Software, San Diego, CA, USA). Each sample was normalized by related β-actin expression or caspase-3 expression. All experiments were repeated three times. *P<0.05, **P<0.01.

We up-regulated and down-regulated HO-1 gene expression in LY-10 cells by using lentivirus-mediated HO-1 transduction and RNA interference respectively, and the mock negative control of target siRNA was used as a scrambled non-targeting sequence [18]. Enhanced green fluorescent protein (EGFP) was detected with fluorescence microscopy (Figure 3A). The apoptosis of LY-10 cells in each group was measured by flow cytometry (FCM) (Supplementary Figure 4E and Supplementary Figure 4F). Treatment with DMSO (0.1%) and lentivirus-mediated HO-1 gene increasing or silencing did not cause LY-10 cell apoptosis (P>0.05). The growth inhibitory effect of SAHA (0.1-12 μM) on LY-10 cells was observed by CCK-8 assay. Such effect increased in time- and concentration-dependent manners. Meanwhile, vector 1 and vector 2 groups had similar inhibited cell proliferation to that of the control group (P>0.05) (Figure 3B). However, the inhibition was significantly increased in the siHO-1 group (P<0.05) (Figure 3C). The cell growth inhibition rate significantly decreased in the HO-1 group (P<0.05) (Figure 3C). Accordingly, lentivirus-mediated HO-1 up-regulation or silencing did not inhibit LY-10 cell proliferation. Nevertheless, silencing HO-1 expression enhanced the effects of SAHA on LY-10 cell proliferation.

### Overexpression of HO-1 protected LY-10 cells from SAHA-induced apoptosis

Compared with control and vector 2 groups, the apoptosis rate of the siHO-1 group significantly increased. In contrast, the apoptosis rate obviously decreased in the HO-1 group compared with that in the vector 1 group (P<0.05) (Figure 3D). In short, HO-1 protein protected against SAHA-induced LY-10 cell apoptosis.

### Silencing HO-1 gene expression enhanced the pro-apoptotic effects of SAHA on LY-10 cells

Cleaved caspase-3 protein increased in the siHO-1 group compared with that in the vector 2 group, and the siHO-1 group had a higher apoptosis rate than that of the vector 2 group (P<0.05). Control and vector 2 groups had similar cleaved caspase-3 protein expressions (P>0.05) (Figure 3H and Figure 3J). However, the apoptosis rate of the siHO-1 group was similar to that of the vector 2 group when treated with SAHA in combination with caspase-3 inhibitor (Z-DEVD) (50 μM) (Figure 3E and Supplementary Figure 4). Taken together, treatment with caspase-3 inhibitor did not cause LY-10 cell apoptosis, and SAHA may induce apoptosis via the caspase-3-dependent pathway.

We also treated LY-10 cells with HO-1 agonist heme (8 μM) and HO-1 inhibitor zinc protoporphyrin (1 μM) in combination with SAHA for 24 h. Western blot was used to detect the expressions of apoptotic proteins (cleaved-PARP and PARP) in LY-10 cells (Supplementary Figure 3). Cleaved-PARP protein, which increased when HO-1 expression was down-regulated, decreased when HO-1 was up-regulated.

### Silencing HO-1 gene expression increased cell cycle arrest (G0/G1) induced by SAHA in LY-10 cells

**Figure 4:**
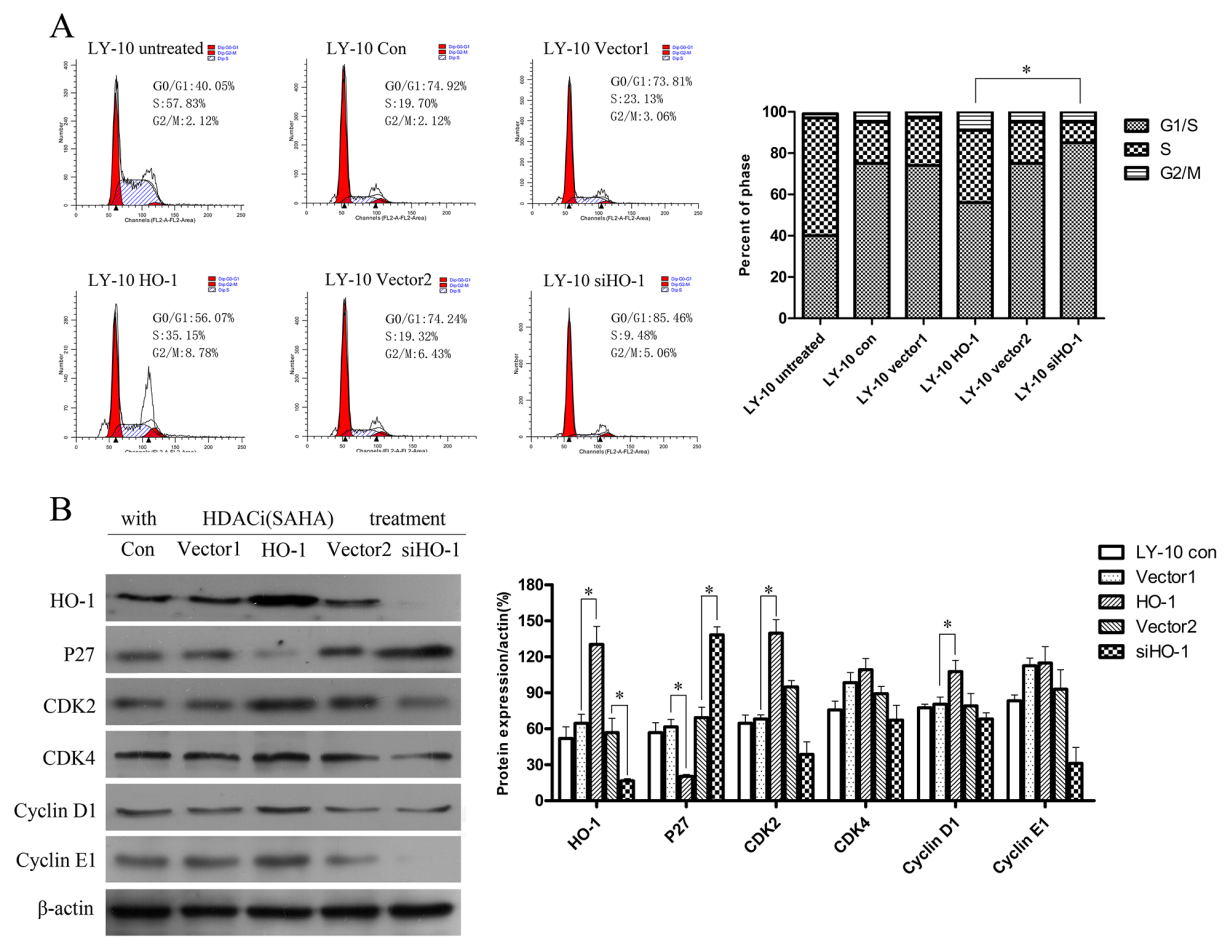
Silencing HO-1 gene expression augmented cell cycle arrest (G0/G1) induced by SAHA in LY-10 cells **(A)** LY-10 cells were grouped into LY-10 (control), LY-10 (vector 1), LY-10 (HO-1), LY-10 (vector 2), and LY-10 (siHO-1). Different groups of LY-10 cells were treated with SAHA (8 μM) for 24 h. Cell cycle was analyzed by flow cytometry. **(B)** Western blot of cell cycle proteins in different groups of LY-10 cells. Protein expressions of HO-1, P27, CDK2, CDK4, cyclin D1, and cyclin E1 were detected. Data were analyzed with Prism V5.0 (GraphPad Software, San Diego, CA, USA). Each sample was normalized by related β-actin. All experiments were repeated three times. *P<0.05, **P<0.01.

LY-10 cells were treated with SAHA for 24 h, and the cell cycle was analyzed by FCM. LY-10 cells in the siHO-1 group were obviously arrested in the G0/G1 phase compared with those in the HO-1 group, accompanied by increased P27^Kip1^ protein expression (P<0.05) (Figure 4A). Furthermore, vector 1 and vector 2 groups had similar P27^Kip1^ protein expressions to that of the control group (P>0.05). However, silencing of HO-1 gene expression combined with SAHA did not significantly increase CDK2 or cyclin E1 protein expression (Figure 4B).

### Silencing HO-1 gene expression altered the ability of SAHA to regulate histone acetylation-related gene expression in LY-10 cells

**Figure 5:**
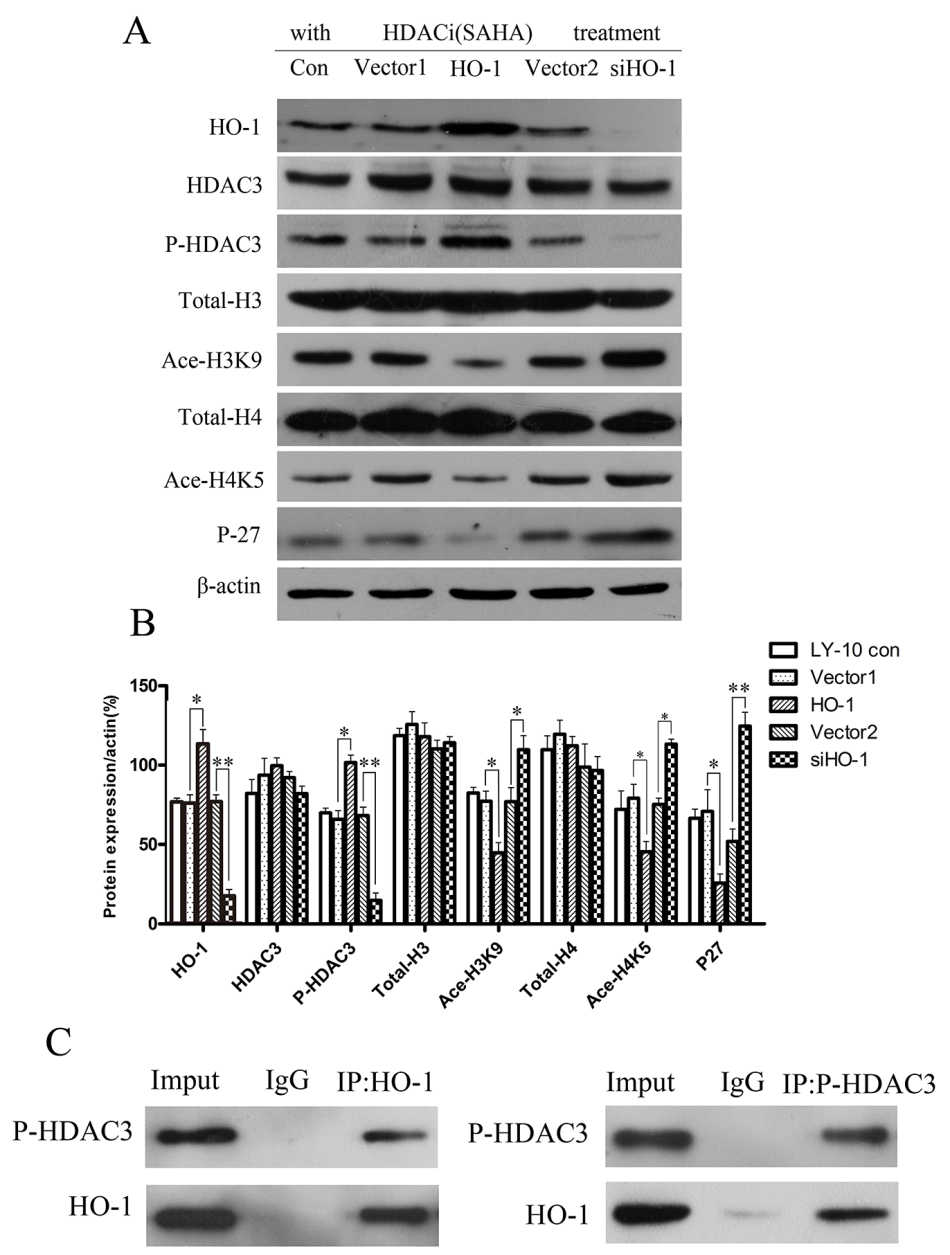
Histone acetylation-related gene expressions in LY-10 cells **(A, B)** LY-10 cells were grouped into LY-10 (control), LY-10 (vector 1), LY-10 (HO-1), LY-10 (vector 2), and LY-10 (siHO-1). Different groups of LY-10 cells were treated with SAHA (8 μM), and histone acetylation-related protein expressions of HO-1, P27, HDAC3, P-HDAC3, total-H3, Ace-H3K9, total-H4, and Ace-H4K5 were analyzed by Western blot. Western blot bands were quantified with Quantity One software. Data were analyzed with Prism V5.0 (GraphPad Software, San Diego, CA, USA). Each sample was normalized by related β-actin expression. All experiments were repeated three times. *P<0.05, **P<0.01.

To analyze the regulatory effects of silencing of HO-1 gene expression combined with SAHA on histone acetylation-related protein expressions in LY-10 cells, we detected HDAC3, phospho-HDAC3 (P-HDAC3), total-H3, acetylated-H3K9 (Ace-H3K9), total-H4, acetylated-H4K5 (Ace-H4K5), and P27^Kip1^ protein expressions in different groups of LY-10 cells by Western blot. Treatment with SAHA significantly increased P-HDAC3 expression compared with that of the vector 1 group (P<0.05), whereas silencing of HO-1 gene expression combined with SAHA decreased P-HDAC3 expression compared with that of the vector 2 group (P<0.05). Ace-H3K9, Ace-H4K5, and P27^Kip1^ protein expressions significantly increased in the siHO-1 group compared with those of the vector 2 group (P<0.01; P<0.05; P<0.01) (Figure 5A and Figure 5B).

In the meantime, co-immunoprecipitation confirmed that HO-1 bound P-HDAC3 while being functionally related (Figure 5C). Collectively, silencing HO-1 gene expression decreased the expression of P-HDAC3 protein but elevated that of P27 ^Kip1^ protein in LY-10 cells.

### Silencing HO-1 gene expression activated histone acetylation of P27^Kip1^ promoter in LY-10 cells

**Figure 6:**
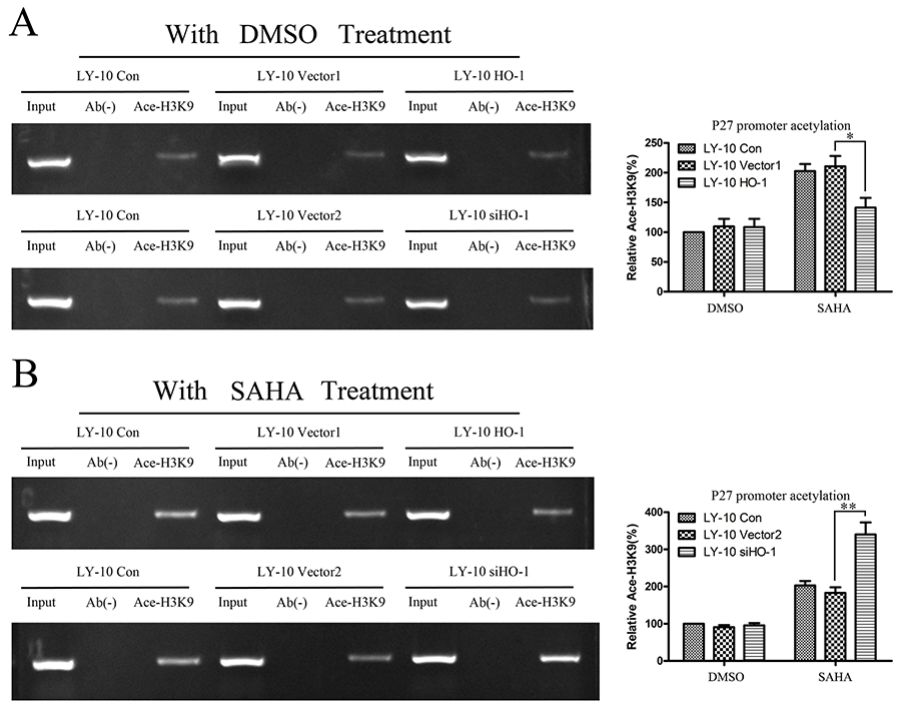
Histone acetylation level of P27^Kip1^ promoter in LY-10 cells **(A, B)** LY-10 cells were grouped into LY-10 (control), LY-10 (vector 1), LY-10 (HO-1), LY-10 (vector 2), and LY-10 (siHO-1). Different groups of LY-10 cells were treated with SAHA (8 μM) or DMSO (0.1%). CHIP-PCR was employed to identify changes in the histone acetylation H3K9 levels at P27^Kip1^ promoter in different groups of LY-10 cells. P27^Kip1^ promoter sequences in the input DNA that was recovered from antibody-bound chromatin segments were quantified by PCR and normalized to corresponding input controls. P27^Kip1^ promoter histone acetylation was examined by agarose gel electrophoresis and quantified with Quantity One software. Data are representative of at least three independent experiments. *P<0.05, **P<0.01.

Chromatin immunoprecipitation (CHIP)-PCR analysis was used to determine the histone acetylation of P27^Kip1^ promoter (Table 3), as an index of its activity in LY-10 cells. As shown in Figure 6, silencing of HO-1 gene expression combined with SAHA can increase P27^Kip1^ promoter Ace-H3K9 level compared with that of the vector 2 group (P<0.01). Hence, silencing HO-1 activated the histone acetylation of P27^Kip1^ promoter in LY-10 cells (Figure 9).

**Table 3:** The primer sequences used in CHIP-PCR

Gene name	Sequences F (5’–3’)	Sequences R (5’–3’)	Length ( bp )
P27	5′-CCTGCTCATCGTCCTACTTT-3′	5′-CCAGATTTCACTGCTCCAAC-3′	254

**Figure 7:**
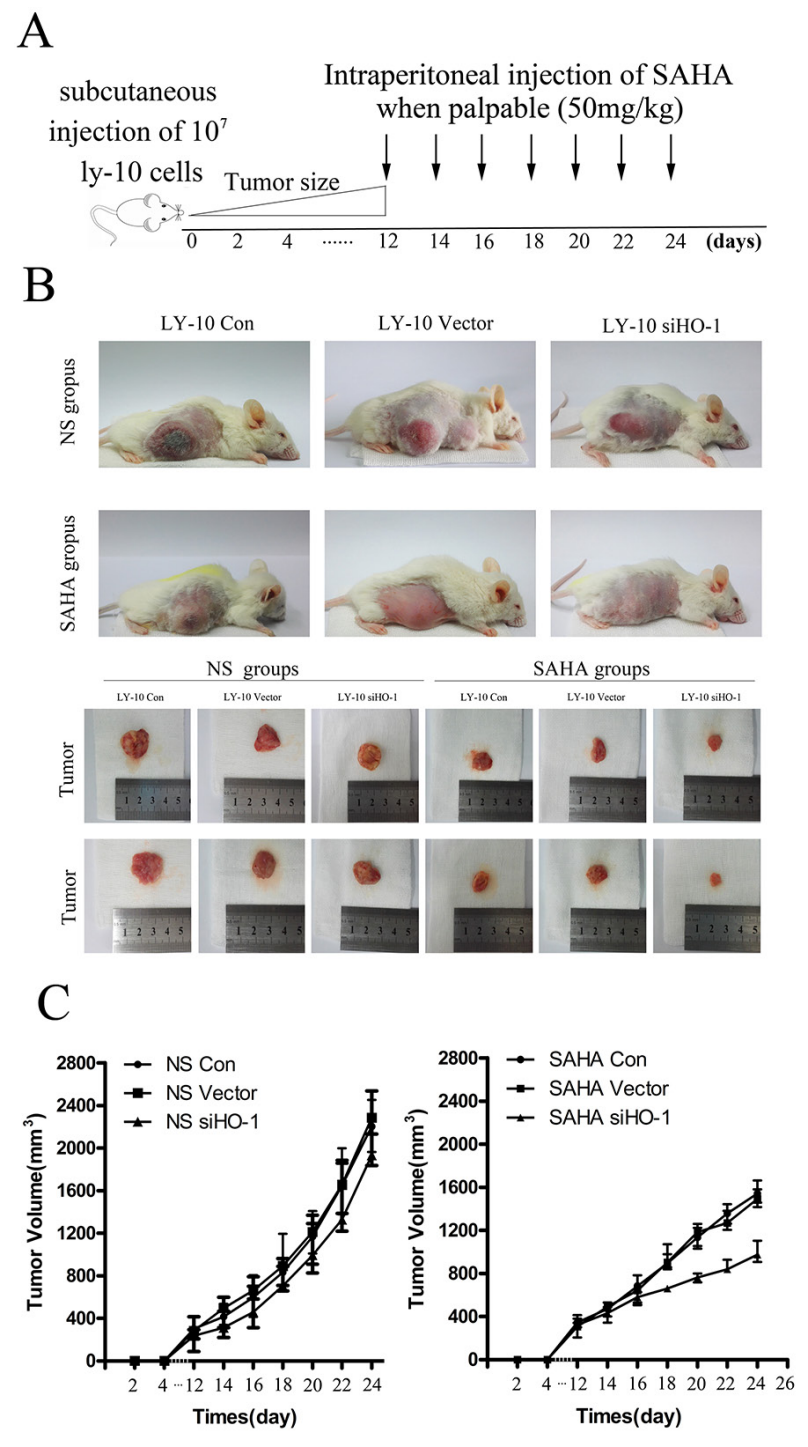
Silencing HO-1 gene expression sensitized SAHA to induce tumor suppression in a xenograft mouse model **(A, B)** We subcutaneously inoculated NOD/SCID mice in both flanks with LY-10 cells (1×10^7^ cells) to establish a xenograft mouse model of DLBCL. Mice were treated every day with 50 mg/kg SAHA when tumors were palpable (day 12). This model was euthanized on the 14th day after treatment with SAHA. Tumor weights and volumes were measured and calculated. **(C)** We used a ruler to measure sizes and weights of spleens from this model. Data are representative of three independent experiments. *P<0.05, **P<0.01.

**Figure 8:**
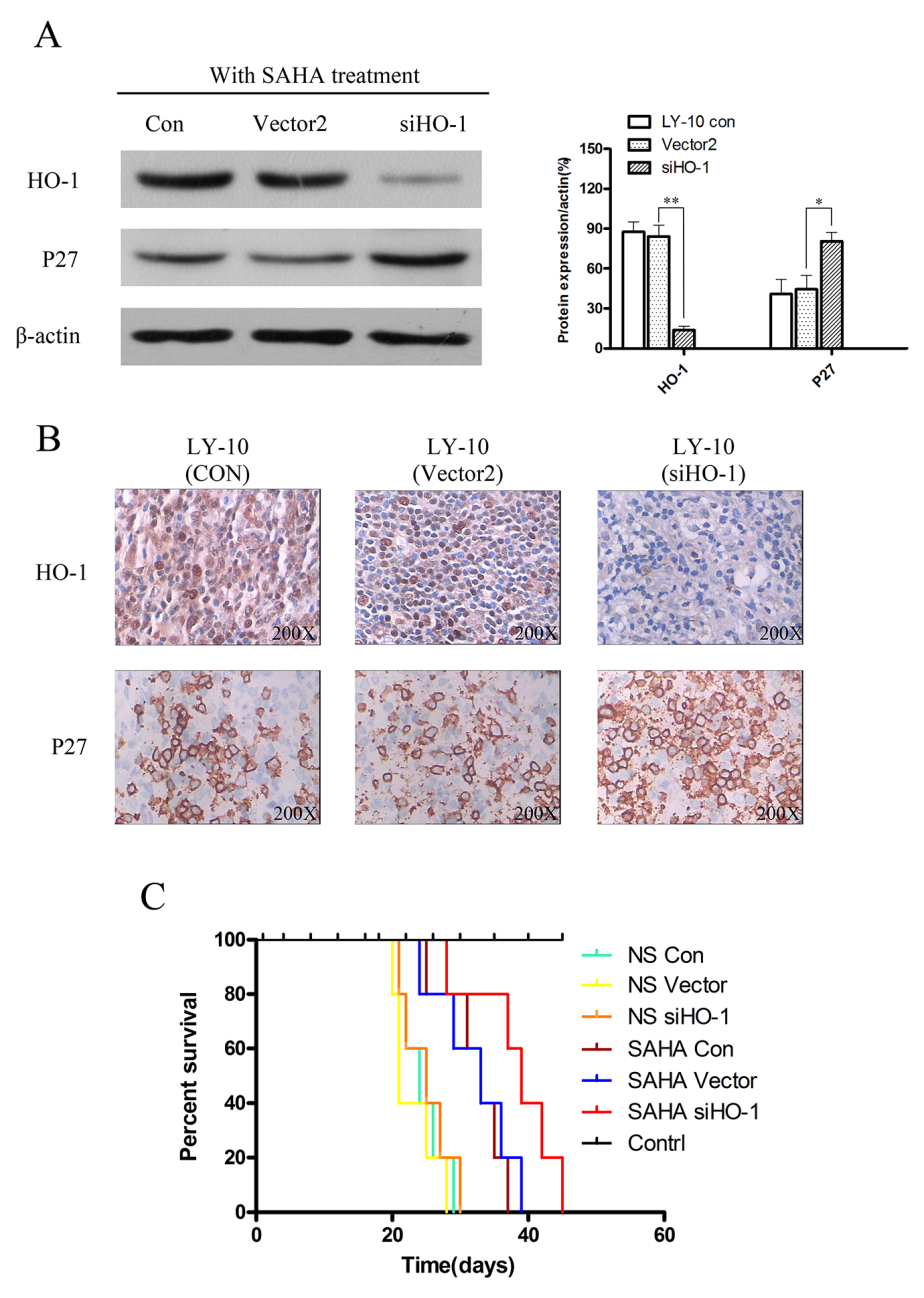
Silencing HO-1 gene expression potentiated SAHA to induce tumor proliferation inhibition and prolonged survival time in xenograft mouse model **(A)** Expressions of HO-1 and P27 in xenograft mouse model tumor tissue were detected by Western blot. Each sample was normalized by related β-actin. All experiments were repeated three times. *P<0.05, **P<0.01. **(B)** HO-1 and P27 in tumor tissue of xenograft mouse model were assessed at the protein level by immunohistochemistry. A representative example (tumors number=6) is shown (400 ×). **(C)** Kaplan-Meier survival curve of each group was plotted from the first day of treatment until death.

**Figure 9:**
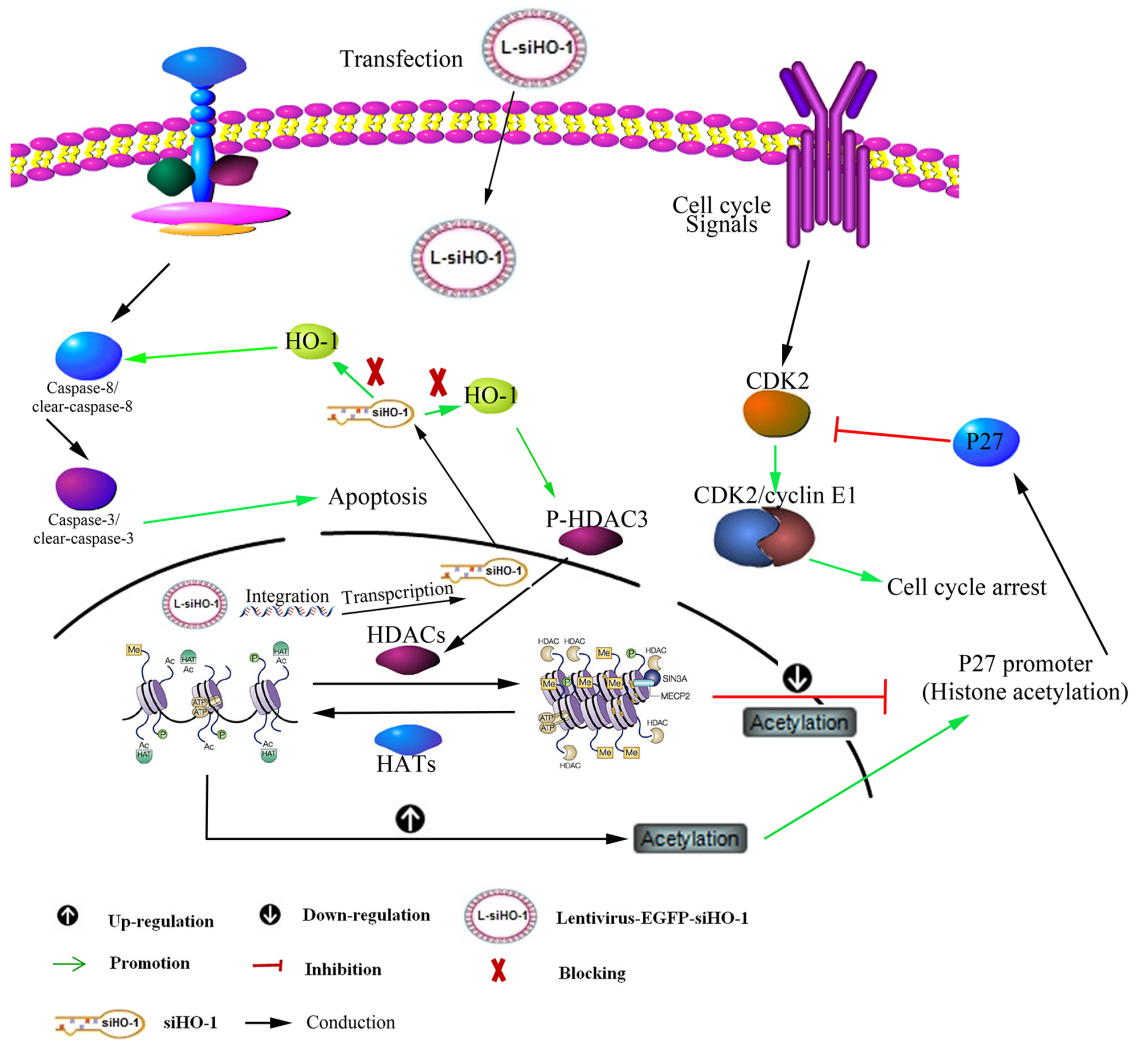
Schematic representation of mechanism for HO-1 silencing associated with SAHA-induced LY-10 cell apoptosis Silencing HO-1 gene expression potentiated SAHA to increase cleaved caspase-3 and tumor suppressor P27 protein expressions and to decrease P-HDAC3 expression. Conversely, overexpression of HO-1 gene reversed these effects.

### Silencing HO-1 gene expression potentiated SAHA to induce proliferation inhibition *in vivo*

Compared with SAHA-treated groups, the control group of xenograft DLBCL mice treated with normal saline developed tumors more rapidly (Figure 7A). Additionally, the growth of tumors in the siHO-1 group was significantly slower than that of the vector 2 group (Figure 7B).

Moreover, P27^Kip1^ protein expression increased in the siHO-1 group compared with that of the vector group induced by SAHA (P<0.05) (Figure 8A and Figure 8B). The overall survival of SAHA-treated model DLBCL mice was prolonged, especially in the siHO-1 group (compared with the normal saline-treated vector group) (P<0.05) (Figure 8C). Treatment with normal saline gave similar results in Con, vector and siHO-1 groups (P>0.05) (Figure 8C).

### Schematizes the mechanism for HO-1 silencing

Figure 9 Treatment with HDACi (vorinostat) may up-regulate HO-1 expression via the NF-κB pathways. Silencing HO-1 may contribute to SAHA-induced apoptosis and arrest cell cycle by activating caspase-3 activity, down-regulating the expression of P-HDAC3 and increasing that of P27 protein in LY-10 cells.

## DISCUSSION

HDACis have anticancer effects on a wide range of malignancies, especially hematological cancers [32]. They are well tolerated in clinical trials and selective to tumor cells [33]. In preclinical experiments, HDACi has anti-proliferative and pro-apoptotic effects on DLBCL cell lines [34–36]. However, single-agent vorinostat is ineffective for relapsed DLBCL patients in clinical practice [29, 37]. The failure of different chemotherapeutic drugs has been attributed to the adaptation of tumor cells to oxidative stress by eliminating reactive oxygen species and toxic molecules [38, 39]. In our previous study, HO-1 protein expression was significantly higher in relapsed and high-risk DLBCL patients than that in normal lymph nodes. In this study, HO-1 protein expression was positively correlated with IPI classification and HDAC3 protein expression, and negatively correlated with P27^Kip1^ expression. HO-1 was highly expressed in LY-3 and LY-10 cells *in vitro* compared with those in LY-7 and LY-19 cells. Furthermore, SAHA treatment increased HO-1 expression by up-regulating phospho-IκB-α^S32/S36^ protein expression and activating the NF-κB pathway in LY-10 cells, exerting a cytoprotective effect. It has also been reported that SAHA increased NF-κB activity [29–31]. Therefore, HO-1 was an anti-apoptotic molecule in DLBCL cell lines and patients.

Subsequently, we used lentivirus to down-regulate HO-1 gene expression in LY-10 cells to investigate the possible mechanism by which high HO-1 expression affected the influence of SAHA on proliferation, apoptosis and cell cycle arrest in the G0/G1 phase. Apoptosis and cell cycle arrest were drastically enhanced by HO-1 silencing but diminished when HO-1 was up-regulated. Likewise, HO-1 overexpression plays a crucial anti-apoptotic role and leads to drug resistance in hematological malignancies such as DLBCL, MM, and AML [18, 40–42]. Moreover, silencing HO-1 gene expression increased LY-10 cell apoptosis induced by SAHA and augmented the expressions of cleaved caspase-3 and cleaved-PARP proteins, which were reversed by caspase-3 inhibitor. Therefore, HO-1 may affect the caspase-3 pathway to promote LY-10 cell apoptosis. Wang et al. also reported that silencing HO-1 gene expression sensitized tumor cell apoptosis via the caspase-3-dependent pathway in MDS [25]. Yet, it is necessary to investigate the effects of HO-1 expression on other apoptotic proteins (e.g. NOXA and MCl-1) in ABC-DLBCL cells.

Silencing of HO-1 gene expression in combination with SAHA facilitated the protein expression of P27^Kip1^, promoting cell cycle arrest in the G0/G1 phase. Meanwhile, silencing HO-1 gene expression enhanced P27^Kip1^ promoter histone acetylation induced by SAHA. Consistently, HDACi can increase the acetylation of histones H3 and H4, leading to increased P27^Kip1^ expression in human neuroblastoma and CML cell lines [43]. Moreover, up-regulating HO-1 protein expression induces up-regulation of P-HDAC3 protein expression, which was reversed by silencing HO-1 gene expression. Similarly, HO-1 protein can bind P-AKT protein and prevent it from degradation [20]. Thus, HO-1 protein bound P-HDAC3 protein as a complex to avoid its degradation, and the activity of HDAC3 protein enhanced P27^Kip1^ promoter acetylation, thereby increasing P27^Kip1^ transcription and protein expression (Figure 9). However, it is necessary to further confirm the results by using HO-1 gene knockout mice.

Silencing HO-1 gene expression efficiently enhanced the effects of SAHA chemotherapy *in vivo*, significantly prolonging survival time and increasing P27^Kip1^ protein expression. Hence, silencing HO-1 gene expression enhanced SAHA-induced apoptosis and arrested cell cycle in the G0/G1 phase.

In conclusion, HO-1 is a potential epigenetic therapeutic target. The findings may provide a valuable preclinical evidence for increasing the sensitivity of ABC-DLBCL patients to HDACi.

## MATERIALS AND METHODS

### Patient samples

According to the World Health Organization classification of lymphatic hematopoietic tissue tumor (2008), we collected five cases of normal lymph nodes and 50 cases of DLBCL (ABC-DLBCL: 42 cases; GCB-DLBCL: 8 cases), with available formalin-fixed paraffin-embedded (FFPE) samples from Guizhou Medical University from January 2010 to December 2015 (Table 1). According to the IPI classification for DLBCL patients, we divided the cases into four different risk groups [44, 45].

### Immunohistochemistry

Lymphoma cells from DLBCL patients were made into FFPE samples and routinely processed by immunohistochemical staining for HO-1, HDAC3, and P27 (HO-1 concentration 1:400, P27 concentration 1:500, heat-induced antigen retrieval, BD Pharmingen, San Jose, CA, USA). According to staining intensity, HO-1, HDAC3, and P27 protein expressions in tumor cells were classified into grades 1 to 3 (weak, intermediate, and strong), and also classified by proportion of stained tumor cells into grades 1 to 4 (1 represents 1–25% of positive tumor cells and 4 represents 75–100% of positive tumor cells) [18, 46].

### Cells and cell culture conditions

Human DLBCL cell lines LY-3, LY-7, LY-10, and LY-19 were cultured in RPMI-1640 medium supplemented with 15% fetal bovine serum, 100 U/mL penicillin, and 100 mg/mL streptomycin [18, 26]. Cell lines were purchased from the China Academy of Shanghai Cell Bioresources. The medium and antibiotics were purchased from Invitrogen (Carlsbad, CA, USA). All cells were maintained in a 37°C incubator with 95% humidity and 5% CO_2_.

### Lentiviral vector and transduction

Construction of recombinant lentiviral vector and transduction were performed using recombinant lentivirus-HO-1-EGFP (HO-1) and its control vector, lentivirus-EGFP (vector 1); and recombinant lentivirus-RNAi-EGFP-siHO-1 (siHO-1) and its control vector, lentivirus-RNAi-EGFP (vector 2). Both pairs of lentiviral vectors were co-transduced into LY-10 cells. The transduction rate was determined using microscopy (Olympus, Tokyo, Japan) and Western blot [18, 22, 25, 26].

### Chemicals

SAHA (99.0% purity) was purchased from Selleckchem (Houston, TX, USA). Z-DEVD-FMK (90.0% purity) was purchased from Abcam (Cambridge, UK). DMSO (99.9% purity) was purchased from Solarbio (Beijing, China).

### Cell viability assay

Different groups of LY-10 cells were seeded at the density of 5000 per well in 96-well plates. The inhibitory effects of SAHA on LY-10 cells were determined using the CCK-8 assay [18].

### Apoptosis analysis

Apoptotic cells were measured using FCM with PI and Annexin-V staining (BD Biosciences, San Jose, CA, USA), and analyzed by Cell Quest software (BD Biosciences) [18].

### Cell cycle analysis by FCM

Cell cycle was measured using FCM with PI staining (BD Biosciences, San Jose, CA, USA). The stained cells were analyzed for DNA content by FCM, and cell cycle phase distributions were analyzed with Cell Quest Pro software (BD Biosciences) [22].

### Western blot

Protein expression was detected by Western blot. Primary antibodies such as HO-1 and HDAC3 were obtained from Santa Cruz Biotechnology (CA, USA) [18, 22, 26]. Phosphorylated primary antibodies such as phospho-IκB-α^S32/S36^ and total-IκB-α were obtained from Cell Signalling Technology (Beverly, MA, USA). Secondary antibodies were obtained from Santa Cruz Biotechnology (CA, USA) and Cell Signalling Technology (Beverly, MA, USA). Equal amounts of protein lysate were used for Western blot. β-Actin expression was consistent in all cases, and then cleaved caspase-3 was compared to caspase-3 protein or phospho-IκB-α was compared to total-IκB-α protein.

### Co-immunoprecipitation assay

Immunoprecipitates captured with Sepharose beads were washed four times with RIPA buffer and analyzed by immunoblotting [27].

### Chromatin immunoprecipitation (CHIP) assay

To measure the amount of acetylated histone H3 bound to P27^Kip1^ promoter, a CHIP assay kit (Upstate Biotechnology) was used according to manufacturer’s instructions [47]. The p27^Kip1^ promoter DNA was amplified by PCR using the primer sequences shown in Table 3. The data obtained were normalized to the corresponding DNA input control.

### Xenograft mouse model of DLBCL

NOD/SCID mice purchased from Beijing Laboratory Animal Center were injected intraperitoneally with 100 mg/kg cyclophosphamide (Wako Pure Chemical Industries, Kyoto, Japan) on 2 days in a row to repress residual immunity. On withdrawal of cyclophosphamide after 2 days, the mice were randomized into three groups, and LY-10 (without treatment), LY-10 vector (transduced with empty vector), and LY-10-siHO-1 (transduced with siRNA targeting HO-1) cells respectively (1×10^7^ cells per animal were injected subcutaneously into the right abdomen) [26]. On the 12th day after inoculation, the three groups were further divided into six groups: LY-10, LY-10-vector, LY-10-siHO-1, LY-10 (SAHA), LY-10-vector (SAHA), and LY-10-siHO-1 (SAHA). Each group of mice consisted of five animals. The mice were administered with SAHA (50 mg/kg) or normal saline intraperitoneally once a day from 12 days onwards. Tumor sizes were measured by vernier calliper twice a day and calculated by π/6 length × width^2^. The survival times of the mice were recorded and analyzed. All procedures were conducted in accordance with Guidelines for the Care and Use of Laboratory Animals. The protocol was approved by the Committee on the Ethics of Animal Experiments of Guiyang Medical University.

### Statistical analysis

Each experiment or assay was performed at least three times, and representative examples were shown. Data were reported as means ± SEM. Statistically significant differences between the treated groups were calculated using the Student’s t-test. Differences were considered statistically significant at P<0.05.

## SUPPLEMENTARY MATERIALS FIGURES


