# Supplementary material for: Silencing heme oxygenase-1 increases the sensitivity of ABC-DLBCL cells to histone deacetylase inhibitor in vitro and in vivo
Source: Oncotarget. 2017 Jul 28;8(45):78480–95. doi: 10.18632/oncotarget.19652 (PMC5667976; doi:10.18632/oncotarget.19652)
Supplement: Supplementary file 1 [file oncotarget-08-78480-s001.pdf]

Silencing heme oxygenase-1 increases the sensitivity of ABC-DLBCL cells to histone deacetylase inhibitor *in vitro* and *in vivo*

SUPPLEMENTARY MATERIALS

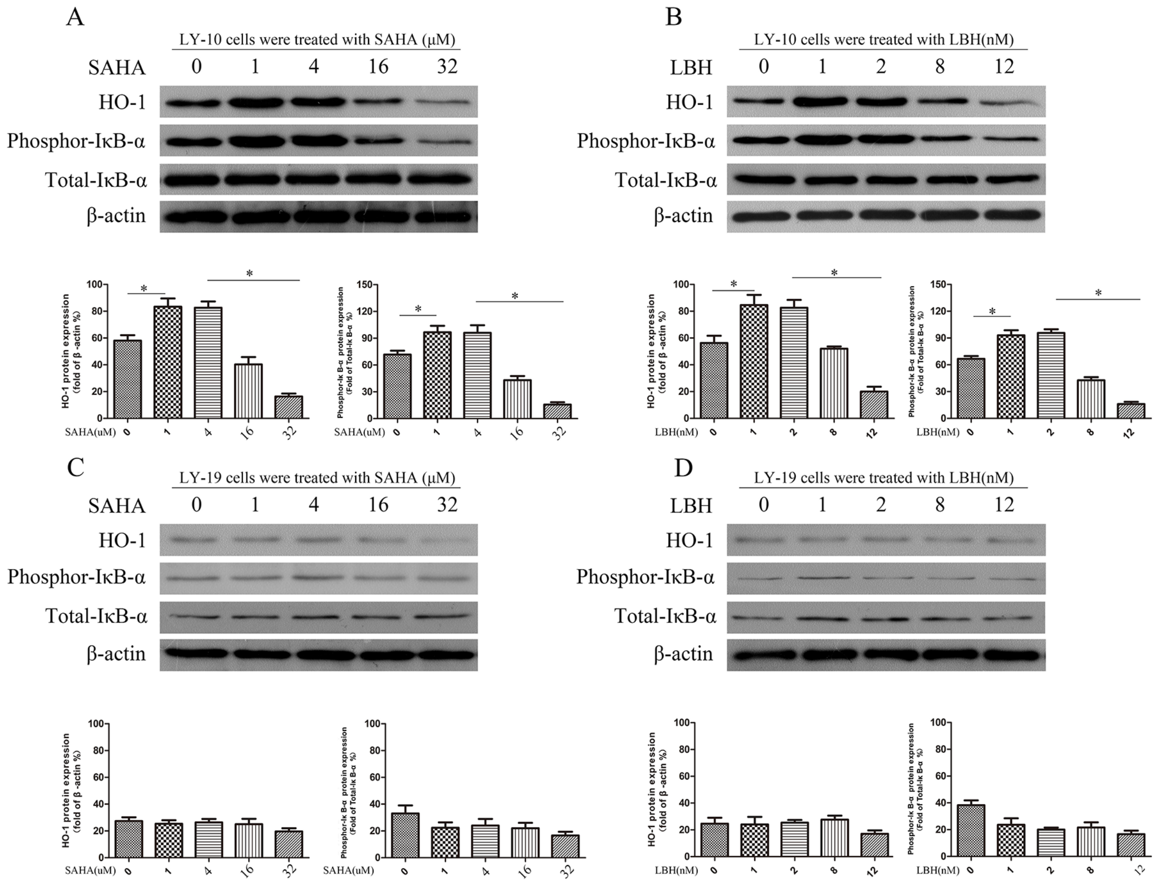

Supplementary Figure 1: Vorinostat and panobinostat treatment affected NF-κB pathway in LY-10 and LY-19 cells. (A, C) LY-10 and LY-19 cells were treated with vorinostat (SAHA) (1, 4, 16 and 32 μM) for 24 h. Protein expressions of HO-1, phospho-IκB-$\alpha^{S32/S36}$, and total-IκB-α were detected by Western blot. **(B, D)** LY-10 and LY-19 cells were treated with panobinostat (LBH) (1, 2, 8, and 12 nM) for 24 h. Protein expressions of HO-1, phospho-IκB-α, and total-IκB-α were detected by Western blot. Western blot bands were quantified with Quantity One software. Each sample was normalized by related β-actin expression or IκB-α expression. All experiments were repeated three times. *P<0.05, **P<0.01.

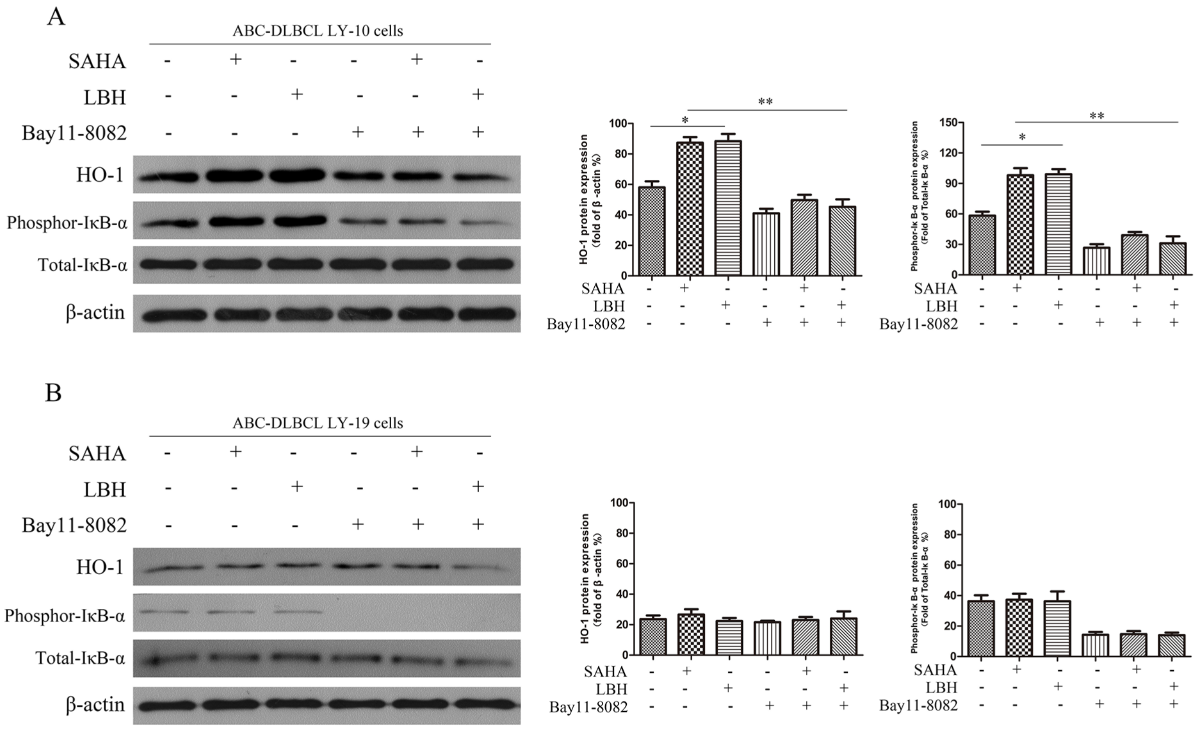

Supplementary Figure 2: NF-κB inhibitor Bay11-7082 attenuated NF-κB activation mediated by HDACis. (A, B) LY-10 and LY-19 cells were pretreated with NF-κB inhibitor Bay11-7082 (6 μM) for 1 h and then treated with vorinostat (SAHA) (4 μM) and panobinostat (LBH) (12 nM) for 24 h. Protein expressions of HO-1, phospho-IκB-α, and total-IκB-α were detected by Western blot. Western blot bands were quantified with Quantity One software. Each sample was normalized by related β-actin expression or total-IκB-α expression. All experiments were repeated three times. *P<0.05, **P<0.01.

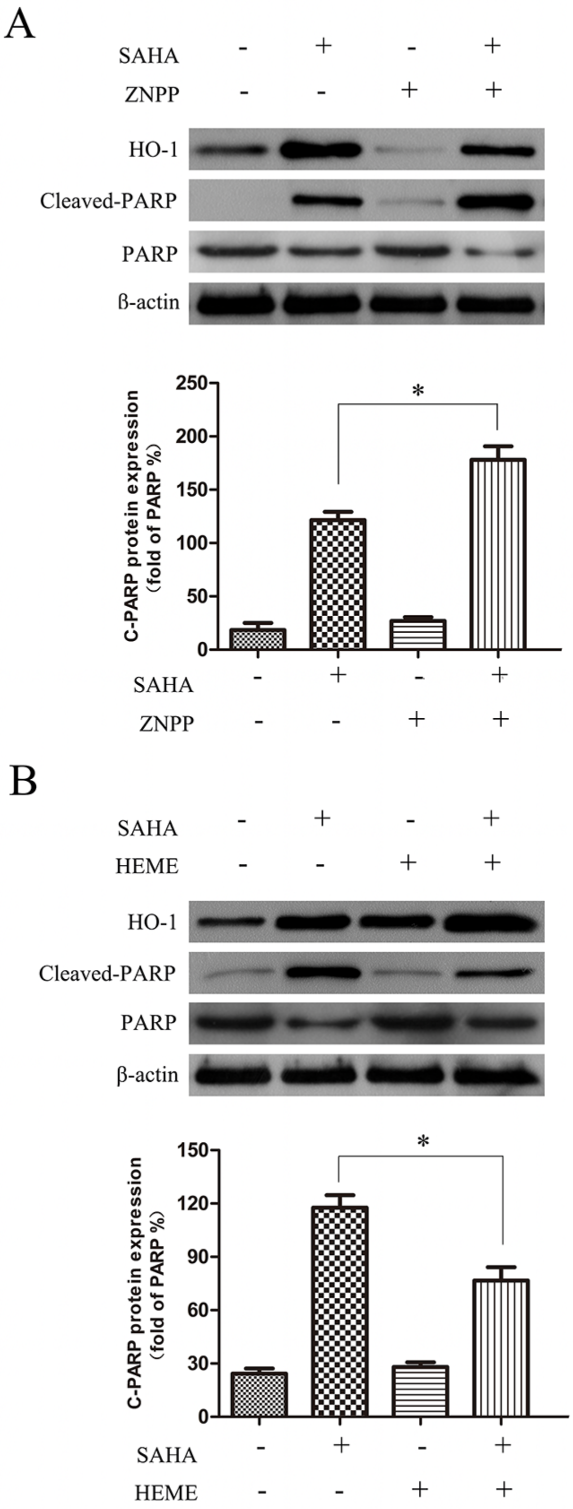

Supplementary Figure 3: Effects of heme or zinc protoporphyrin on SAHA-regulated apoptosis proteins in LY-10 cells. (A, B) LY-10 cells were treated with vorinostat (SAHA) (8 μM) combined with HO-1 protein inhibitor zinc protoporphyrin (1 μM) or HO-1 protein activator heme (8 μM) for 24 h. Protein expressions of HO-1, cleaved-PARP, and PARP were detected by Western blot. Western blot bands were quantified with Quantity One software. Each sample was normalized by related PARP expression. All experiments were repeated three times. *P<0.05, **P<0.01.

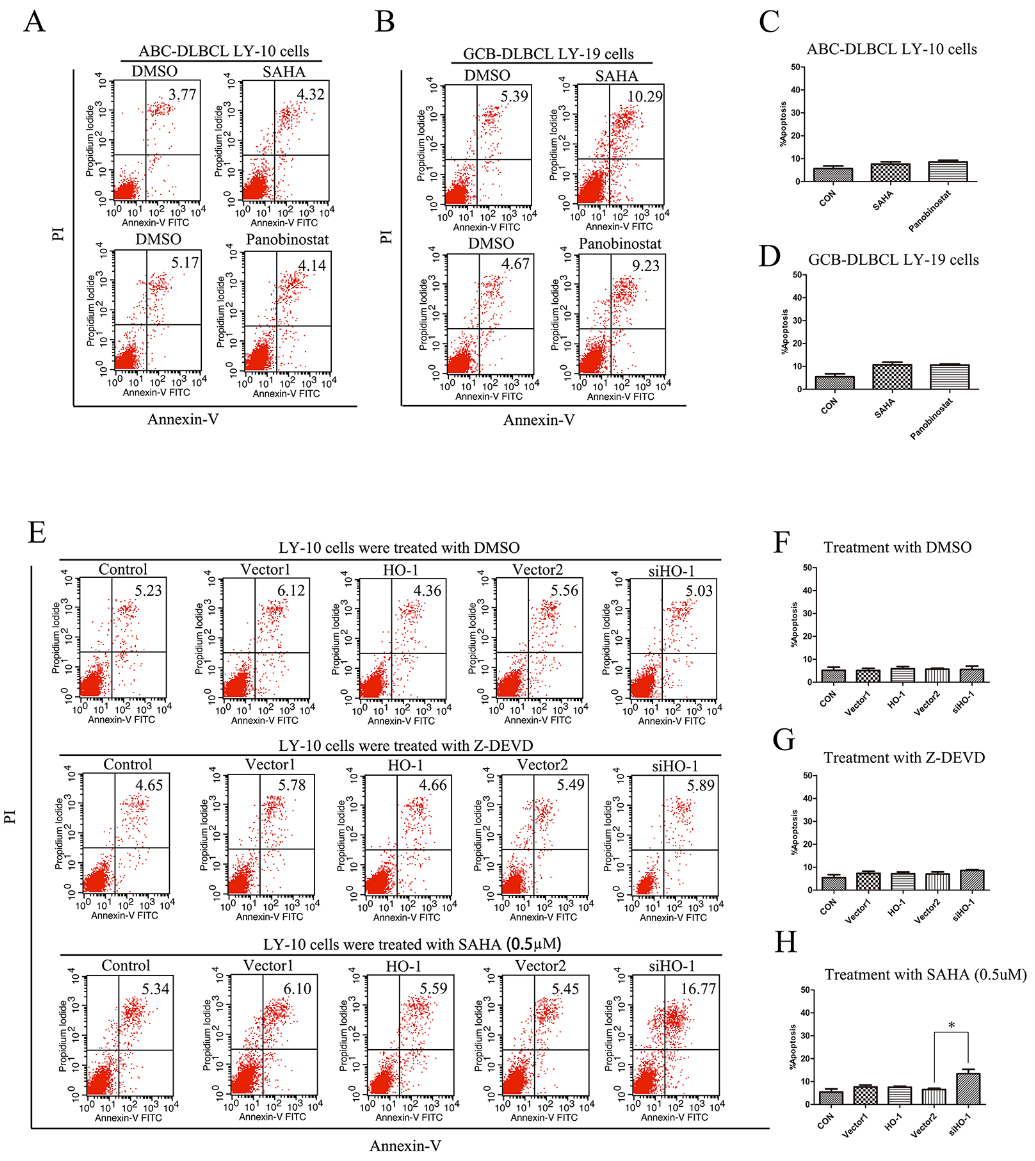

Supplementary Figure 4: Apoptosis rates of LY-10 and LY-19 cells. (A, B) LY-10 and LY-19 cells were treated with vorinostat (SAHA) (0.5 μM), panobinostat (1 nM) and DMSO (0.1%) for 24 h. Apoptosis rate was detected by flow cytometry. **(E)** LY-10 cells were treated with DMSO (0.1%), Z-DEVD (50 μM) and SAHA (0.5 μM) for 24 h. Apoptosis rate was detected by flow cytometry. **(C, D, F, G, H)** Graphs show apoptotic LY-10 or LY-19 cells in each group. Data were analyzed with Prism V5.0 (GraphPad Software, San Diego, CA, USA). All experiments were repeated three times. *P<0.05, **P<0.01.
